# Health numeracy in Japan: measures of basic numeracy account for framing bias in a highly numerate population

**DOI:** 10.1186/1472-6947-12-104

**Published:** 2012-09-11

**Authors:** Masako Okamoto, Yasushi Kyutoku, Manabu Sawada, Lester Clowney, Eiju Watanabe, Ippeita Dan, Keiko Kawamoto

**Affiliations:** 1 Research Center for Animal Hygiene and Food Safety, Obihiro University of Agriculture & Veterinary Medicine, Inada-cho, Obihiro, Hokkaido 080-8555, Japan; 2 Functional Brain Science Laboratory, Center for Development of Advanced Medical Technology, Jichi Medical University, 3311-1 Yakushiji, Shimotsuke, Tochigi 329-0498, Japan; 3 Department of Agro-Environmental Science, Obihiro University of Agriculture and Veterinary Medicine, Inada-cho, Obihiro, Hokkaido 080-8555, Japan

**Keywords:** Risk communications, Patient empowerment, Patient education, Risk perception, Decision making

## Abstract

**Background:**

Health numeracy is an important factor in how well people make decisions based on medical risk information. However, in many countries, including Japan, numeracy studies have been limited.

**Methods:**

To fill this gap, we evaluated health numeracy levels in a sample of Japanese adults by translating two well-known scales that objectively measure basic understanding of math and probability: the 3-item numeracy scale developed by Schwartz and colleagues (the Schwartz scale) and its expanded version, the 11-item numeracy scale developed by Lipkus and colleagues (the Lipkus scale).

**Results:**

Participants’ performances (n = 300) on the scales were much higher than in original studies conducted in the United States (80% average item-wise correct response rate for Schwartz-J, and 87% for Lipkus-J). This high performance resulted in a ceiling effect on the distributions of both scores, which made it difficult to apply parametric statistical analysis, and limited the interpretation of statistical results. Nevertheless, the data provided some evidence for the reliability and validity of these scales: The reliability of the Japanese versions (Schwartz-J and Lipkus-J) was comparable to the original in terms of their internal consistency (Cronbach’s α = 0.53 for Schwartz-J and 0.72 for Lipkus-J). Convergent validity was suggested by positive correlations with an existing Japanese health literacy measure (the Test for Ability to Interpret Medical Information developed by Takahashi and colleagues) that contains some items relevant to numeracy. Furthermore, as shown in the previous studies, health numeracy was still associated with framing bias with individuals whose Lipkus-J performance was below the median being significantly influenced by how probability was framed when they rated surgical risks. A significant association was also found using Schwartz-J, which consisted of only three items.

**Conclusions:**

Despite relatively high levels of health numeracy according to these scales, numeracy measures are still important determinants underlying susceptibility to framing bias. This suggests that it is important in Japan to identify individuals with low numeracy skills so that risk information can be presented in a way that enables them to correctly understand it. Further investigation is required on effective numeracy measures for such an intervention in Japan.

## Background

Active involvement of patients in decision making for their own medical care, such as deciding whether or not to accept a particular treatment (informed consent), or choosing among medical-care options (informed choice), is becoming a worldwide practice [1]. In Japan, informed consent was codified in the “Medical Service Act” in 1997, and is now common practice [2]. Recent surveys show that moving beyond informed consent, Japanese patients are involved in informed decision making in clinical practice [3] and that they prefer this involvement [4]. Consequently, it is very important to ensure that patients correctly understand medical information, so that their decisions, possibly made in life-threatening situations, reflect their true will.

One way to address this issue would be to assess patients’ health numeracy, the ability to understand probabilistic and mathematical concepts [5]. Health numeracy has gained increasing attention, as the amount of quantitative information, such as the probability of survival outcomes for different medical treatments, is increasingly present in medical risk information [6,7]. Previous studies assessing health numeracy have shown that individuals with low numeracy are more likely to misunderstand risk information, and their risk evaluations tend to be more influenced by context, such as how the related numbers are framed (reviewed in [5,8-11]). While many of those studies used healthy respondents, studies with actual patients have shown the influence of numeracy on their disease-related decision making (e.g. [12-15]). Therefore, to ensure accurate medical-risk communication, it is important to know whether patients have a sufficient level of health numeracy.

Despite its importance, however, health numeracy and assessment scales have been largely understudied in many countries, including Japan. In fact, previous research is mostly from the United States and Europe, with research in other countries just beginning (e.g. [16-18]). These pioneering studies on cross-cultural comparisons have shown considerable differences in numeracy levels [16,19], as well as the association of numeracy with decision making [20,21] across different countries. Therefore, it is important to determine how previous findings apply to different countries, and develop strategies suitable for each case.

In Japan, the limited attention paid to health numeracy might be a reflection of the common belief that the majority of Japanese people enjoy basic numeracy. For example, since its inception in 2000, the Programme for International Student Assessment has rated the mathematical ability of Japanese students (aged 15 to 16 years) as higher than the international average, whereas that in the United States has been below average. While this might imply that Japanese patients are able to correctly use numerical medical data, recent pioneering work in Japan suggests this might not be the case [22]. In their study, Takahashi et al. developed a 7-item scale to test the ability of Japanese patients to interpret medical information (TAIMI), with 3 of these items especially relevant to numeracy. Unexpectedly, more than 50 percent of respondents made mistakes in 2 of these items. Those 2 items evaluated the effect of a medicine, one presenting information as a fraction and the other as a natural frequency. This result suggests that Japanese health numeracy may not be as high as expected, and that there is a need for further investigation specifically focusing on health numeracy.

The aim of this study is twofold: 1) to evaluate Japanese versions of numeracy scales, and 2) to assess the health numeracy of Japanese adults.

We chose two well-known health numeracy scales that focus on the basic understanding of math and probability, the 3-item Schwartz scale [23], and its expanded version, the 11-item Lipkus scale [24]. To date, these scales are among the most frequently used instruments in numeracy studies. Since the focus of these tests is different from that of TAIMI, we were interested in how Japanese people performed in these health numeracy scales. Japanese versions of these scales (Schwartz-J and Lipkus-J) were prepared using forward and backward translation procedures [25].

The reliability of the scales was assessed for internal consistency. The original scales were shown to be unidimensional; the original Schwartz study did not conduct a factor analysis, but Lipkus and colleagues evaluated the factor structure of their scale which included the Schwartz items [24]. However, there are also studies showing multi-factor structure on these scales [18]. Since factor structure could be different depending on the nature of a target population [26], an exploratory factor analysis was conducted to explore factor structure for the Japanese version. Convergent validity of numeracy scales was evaluated by their correlation with existing measures of health numeracy [8]. As mentioned above, TAIMI has a two-factor structure where three items are specifically relevant to numeracy [22]. Thus, we examined correlations between TAIMI scores and the Schwartz-J and Lipkus-J scores. We expected a positive correlation between performance on TAIMI and the scales translated in this study, specifically for the numeracy items of TAIMI (TAIMI-num).

To determine whether numeracy levels measured by these scales have any influence on medical risk communication in Japan, we examined the association between framing bias and performance on numeracy scales. Previous studies found that those with low scores on the Lipkus scale are more susceptible to framing effects [12,19,27,28], an effect whereby different phrasing influences participant decisions based on mathematically identical data. We tested whether this can be seen with Japanese samples using the Schwartz-J and Lipkus-J scales. To approximate the Japanese population, we used quota sampling (n = 300) according to age, gender, and education level. In so doing, this study explores a method for assessing health numeracy in the Japanese population, with the aim of improving medical-risk communication.

## Results

### Study population

Participant demographics are summarized in Table 1. Overall, the demographics matched those of the Japanese population: education level, gender, and age group were controlled for during recruitment (Additional file 1: Table S1). While household income was not a controlled factor, it also turned out to be similar to that of the general population (Additional file 2: Table S2).

**Table 1:** **Characteristics of the study sample (n = 300**)

	**n = 300**
Age (range, 20–69: % for each decade)	20
Gender (% women)	50
Education (% low attainment)	55
Household income (million yen^b^, %)	
<3	20
3 ≤ 5	21
3 ≤ 8	32
≥8	18
Hospital use (last year, % occasional or often)	53
TAIMI scores	
all (possible range 0–7, mean ± SD)	4.7 ± 1.7
num (possible range 0–3, median ± IQR)	2.0 ± 2.0

^a^Education, low attainment (high school or lower); high attainment (higher than high school).

^b^1 million yen was about 12,000 U.S. dollars at the time of the study.

### Scale characteristics

#### Reliability

Tetrachoric correlations among the items indicated that items 8 and 9 of Lipkus-J were highly correlated (ρ = .99). Therefore, item 8 (the one with less variance) was removed from the analysis to avoid redundancy. A one-factor structure was indicated for both Schwartz-J and Lipkus-J (Table 2), as was also reported in the original study by Lipkus et al. However, while all the items were accounted for by the first factor in the original study, in our study, one item (item 11) did not produce a sufficient factor loading score for Lipkus-J. Hereafter, we refer to the scale consisting of the 9 items that formed the first factor as Lipkus-J^9^, and mainly focus on this scale when dealing with total scores. The scale, including all 11 items is referred to as Lipkus-J^all^. Cronbach’s α of Schwartz-J (0.53) and Lipkus-J^9^ (0.72) were comparable to those reported by the original developers (Schwartz scale, 0.56-0.80 [8]; Lipkus scale, 0.70 - 0.75 [24]).

**Table 2:** Questionnaire items, correct response rate, and factor loading for each item for Schwartz-J and LipkusJ

**Question**		**Answered correctly**^**a**^	**Schwartz-J**^**c**^		**Lipkus-J**^**d**^	
			**Factor loading**^**b**^	**Communality (h**^**2**^**)**	**Factor loading**^**b**^	**Communality (h**^**2**^**)**
1 Imagine that we rolled a fair, six-sided die 1,000 times. Out of 1,000 rolls,how many times do you think the die would come up even (2, 4, or 6)?		85(55)	**0.66**	0.43	**0.68**	0.46
2 *In a lottery, the chance of winning a 1,000 yen prize is 1%.What is your best guess about how many people would win a 1,000 yen prize if 1,000 people each buy a single ticket to this lottery?		82(60)	**0.74**	0.55	**0.63**	0.40
3 *In a lottery, the chance of winning a car is 1 in 1,000. What percent of tickets to this lottery win a car?		73(21)	**0.66**	0.43	**0.61**	0.37
4 Which of the following numbers represents the biggest risk of getting a disease? 1 in 100, 1 in 1000, 1 in 10		95(78)			**0.90**	0.81
5 Which of the following numbers represents the biggest risk of getting a disease? 1%, 10%, 5%		97(84)			**0.82**	0.67
6 If Person A’s risk of getting a disease is 1% in ten years, and person B’s risk is double that of A’s, what is B’s risk?		92(91)			**0.78**	0.61
7 If Person A’s chance of getting a disease is 1 in 100 in ten years, and person B’s risk is double that of A’s, what is B’s risk?		90(87)			**0.71**	0.50
8 If the chance of getting a disease is 10%, how many people would be expected toget the disease:A: Out of 100?		92(81)				
9 If the chance of getting a disease is 10%, how many people would be expected toget the disease:B: Out of 1000?		91(78)			**0.77**	0.60
10 If the chance of getting a disease is 20 out of 100, this would be the same as having a ____% chance of getting the disease.		93(70)			**0.77**	0.59
11 The chance of getting a viral infection is .0005. Out of 10,000 people, about how many of them are expected to get infected?		69(49)			0.18	0.03

^a^Percentage of participants who responded correctly to each item. Corresponding values on the original American study are shown in parentheses ([15], n = 463).

^b^Factor loadings are shown as pattern matrix.

^c^For Schwartz-J, one factor consisting of all three items accounted for 47.1% of variance.

^d^For Lipkus-J, one factor consisting of 9 items accounted for 55.6% of variance.

*Slightly modified from the original for the Japanese context.

#### Convergent validity

Convergent validity of the scales was indicated by significant positive correlations between the scores of each scale and TAIMI. As expected, the associations were stronger with TAIMI-num, the numeracy component of the TAIMI scale (Spearman’s ρ, with Schwartz-J, 0.42; Lipkus-J^9^, 0.46), than with TAIMI-all (Spearman’s ρ, with Schwartz-J, 0.29; Lipkus-J^9^, 0.33).

### Performance on the numeracy scales

Respondents spent 4.9 min ± 0.3 (mean ± SD) completing the Lipkus-J^all^, which included Schwartz-J. On average, the item-wise correct response rate was nearly 90 percent, which is much higher than that of the American sample reported in the original study [24] (Table 2). This high level of performance indicates a high level of basic numeracy among the Japanese public. In particular, in the comparison of risk section that was presented in the same format (items 4 and 5), more than 95% of responses were correct. However, there were two other items where nearly 30 percent of participants made mistakes. These were conversion of fractions to percentages (item 3), and conversion of percentages to frequencies (item 11), both of which included decimal numbers. This suggests that a significant proportion of Japanese people become confused when dealing with such data.

A summary of the total scores for our numeracy measures are listed in Table 3. As in the previous studies using original scales e.g. [28-33], correct responses to Schwartz-J and Lipkus-J^9^ had skewed distributions. Among the demographic characteristics, education level and gender had significant effects on performance on most of the scales, as found in previous American and German studies [33]. In our study, males with high educational attainment performed significantly better than others (see details in Additional file 3: Table S3). The effect of age was significant only in Schwartz-J, and household income did not have any significant effect (Additional file 3: Table S3).

**Table 3:** Total score for each numeracy measure

	**Schwartz-J**	**Lipkus-J**^**9**^	**(Lipkus-J**^**all**^**)**
Mean ± SD	**2.4 ± 0.8**	**8.0 ± 1.5**	9.6 ± 1.8
Median ± IQR	**3.0 ± 1.0**	**9.0 ± 1.0**	10.0 ± 2.0

### Numeracy and framing bias

To examine whether numeracy is an important consideration for medical risk communication in a Japanese sample, we examined the degree of framing bias for two respondent groups - those scoring low, and those scoring high in the Schwartz/Lipkus scales. A median split of the measures (grouping based on Schwartz-J, cut-off score ≤ 2; grouping based on Lipkus-J^9^, cut-off score ≤ 9) was made because, as in previous studies, the distribution of scores for both of these scales was skewed [12,28].

To investigate framing bias, we compared the risk rating scores for a surgical risk for two different framing conditions (survival rate vs. death rate) for each respondent group. In the high-numeracy group, risk ratings did not differ significantly between the two frames (Table 4, Wilcoxon signed rank test, grouping based on Schwartz-J, Z =-0.1, p > 0.9; Lipkus-J^9^ Z =-0.3, p > 0.7). In contrast, participants in the low-numeracy group rated the risk framed as a survival rate as being significantly more risky than the risk framed as a death rate (Table 4, Wilcoxon signed rank test, grouping based on Schwartz-J, Z = −2.7, p < 0.01; Lipkus-J^9^ Z = −3.0, p < 0.01). The framing effect, defined as the difference in riskiness scores between the two frames, was also different between the low- and high-numeracy groups. The degree of framing bias was significantly larger in the low-numeracy group (Mann–Whitney's test, grouping based on Schwartz-J, Z = −2.6, p < 0.01; Lipkus-J^9^, Z = −2.8, p < 0.01). These results indicate that those performing less well on the numeracy scales are more likely to be influenced by how the numerical data are framed, rather than the numbers themselves.

**Table 4:** Difference in framing bias between low and high performance groups for each numeracy measure

**Group (n)**^**a**^	**Schwartz-J**		**Lipkus-J**^**9**^	
	**Low (119)**	**High (181)**	**Low (139)**	**High (161)**
Numeracy score (Median ± IQR)	2 ± 1	3	7 ± 2	9
Difference in rated risk (mean ± SD)^b^	−0.22 ± 0.85	−0.02 ± 0.81	−0.22 ± 0.89	−0.00 ± 0.78

^a^n, number of respondents in each group.

^b^Difference in rated risk was calculated as the difference between survival-rate frame scores and death-rate frame scores. Scores between subgroups were compared using non-parametric methods, but means are presented because median scores did not show differences between sub-groups.

Because numeracy scores were influenced by education and gender, we examined whether these factors also influenced risk perception bias. We did not find significant effects for education (p > 0.5) or gender (p > 0.2) on the framing effect. This suggests that numeracy is a more important determinant of risk perception bias than the demographic characteristics that were examined in the current study.

## Discussion

In this study, we evaluated Japanese numeracy by translating and applying the Schwartz and Lipkus scales, the widely used health numeracy scales that focus on the understanding of basic math and probability. Translated versions of both scales showed certain reliability and validity, however, the Japanese sample’s high performance caused the score distributions to be negatively skewed, imposing limitations on the psychometric evaluations of the scales. In this section, we first discuss Japanese numeracy in light of our results. Then we address the validity and limits of Lipkus-J and Schwartz-J, and future directions for the application of health numeracy measures.

The current study suggests that basic understanding of math and probability is quite high among Japanese: correct response rates for Lipkus-J items were much higher than those found in the original US samples [24], and in more recent studies on probabilistic national German and US samples [33]. This is consistent with the results of the Programme for International Student Assessment (PISA), where the national average math score for Japanese students has been surpassing those of both the US and Germany since its inception [34-37]. The relatively high attainment of math skills during school education, as assessed by PISA, might partly account for the generally high numeracy of the Japanese. The current result is also in line with the recent study assessing the numeracy skills of students at top universities in 15 counties [16]. Although linking top-university level performance with that of the general population is not straightforward, Japan was second best in having the smallest proportion of respondents falling into the lowest quartile.

However, in spite of generally high numeracy, the performance of Japanese sample on the Schwartz-J and Lipkus-J tests still accounted for susceptibility to the framing effect, which can influence patients’ decisions regarding their medical options, such as acceptance of surgery (e.g. [12,19,38]; however, empirical results on framing effects in a clinical setting are mixed, reviewed in [39]). A number of previous studies using the original Schwartz and Lipkus scales have shown a numeracy effect on understanding and decision making based on medical information (reviewed in [5,8-11]). Moreover, studies have been advancing for communicating quantitative risk information with consideration of patients’ numeracy, such as supplementing numerical data with visual or verbal aides, using natural frequencies rather than probabilities, or presenting risks with both negative and positive frames (reviewed in [10,11,40-43]). Considering our results and these earlier findings, such care would be called for when communicating medical information to those with low numeracy in Japan, and possibly in other countries where general math performance is deemed to be high.

Regarding instruments to identify those with low numeracy, both the Schwartz-J and Lipkus-J scales demonstrated certain reliability and validity, with Cronbach’s α being comparable with those of original scales, convergent validity being supported by their positive correlation with other health literacy and numeracy measure (TAIMI, [22]), criterion validity being suggested by their association with the susceptibility to framing bias, and content validity being ensured in the original scales. However, we also found a pronounced ceiling effect, which confounded the analysis we have applied, and limited the psychometric qualities of the scales.

Ceiling effects pose multiple psychometric limitations [16]. First, they suggest that scales are less able to differentiate among those with high numeracy. Second, statistical methods applicable for data analysis become limited, as many popular methods assume a normal distribution, and possibly giving in erroneous results when this assumption is violated [44-46]. Non-parametric alternatives are not always sufficient. For example, in the current study, we had to use a median split, making it difficult to examine the relationship between numeracy scores and framing effects in depth.

A third limitation is that the means to evaluate the validity of the scale. For example, respondents’ performances can be confounded with other factors such as motivation [47]. However, ensuring discriminant validly is not straightforward with data having a ceiling effect, as, for example, a weak correlation between motivation and numeracy scores might be due to the ceiling effect, rather than the variables being truly unrelated. Similarly, examining the relationships between measured ability with other closely related abilities such as working memory [48] would be confounded by the ceiling effect. Thus, use of Lipkus-J and Schwartz-J with high numeracy sample requires careful consideration of those limitations.

In fact, negative skew for the original Schwartz and Lipkus scales have been noted in a number of earlier studies [28-33], and the limitations mentioned above have been pointed out [16,27]. In response to those concerns, new numeracy scales have recently been developed: the Berlin Numeracy Test (BNT, [16]) and the Abbreviated Numeracy Scale (ANS, [27]). While both scales were built on the works of Schwartz and Lipkus, they have a wider range of difficulty. As a result, they have better psychometric characteristics, especially when used with high-performance samples. Considering the generally high numeracy of Japanese, those new scales might be more suitable for assessing numeracy in Japan, and this should be explored.

Meanwhile, Lipkus-J and Schwartz-J could be useful for assessing those having low numeracy. In the above-mentioned studies that developed new numeracy scales, the effectiveness of the original Lipkus and Schwartz scales is indicated for assessing groups with low numeracy [16,27]. In fact, positive skew was observed in some of the samples studied using BNT [16], where easier tests would work better. This is an important point to consider when clinical applications are in scope, because some patients are likely to be under physical and psychological stress, which might result in lower numeracy. For instance, a recent clinical study using the Lipkus scale found the numeracy of epilepsy patients to be significantly lower than healthy controls even though educational attainments were lower in the control group [12]. This issue also bears on the test’s validity; where the psychometric characteristics of scales could differ across population groups or settings [49]. Considering possible difference between patients and healthy groups, the use of the numeracy scales translated here, as well as the above-mentioned new scales, should be explored using patient samples so that more effective numeracy measures for the patient population can be discovered.

Finally, the possible influence of volunteer bias [50] should be noted when interpreting the current results. Although demographics of the sample matched those of the Japanese adult population, the test respondents were those who voluntarily agreed to participate in a survey concerning numbers. Therefore, the results could be biased towards those who are more interested in solving numerical problems, and not actually representative of the population. In fact, the average total score of TAIMI in the current study was 4.7, which is higher than that of 3.9 in the original report (Internet survey, n = 6047, [22]). This disparity might be due to differences in sample composition between Takahashi et al.’s work and ours (there were more females and elderly in their study, and no education levels were reported). However, it is also possible that the numeracy reported here is higher than average. This issue should be addressed in future random-selection population-based surveys.

## Conclusions

The current study highlights the importance of considering health numeracy in Japan. As assessed by the Japanese versions of internationally used health numeracy scales, the basic understanding of math and probability by Japanese people was shown to be high, but still not sufficient for many to avoid framing bias. Thus, to improve numerical medical risk communication in Japan, it would be necessary to assess health numeracy, screening those with low numeracy to provide them with appropriate care. Although efforts have been made [16,22], numeracy has not gained much attention in Japan. By evaluating the health numeracy of Japanese, and its measurement instruments, our study is a step towards improving medical risk communication.

## Methods

### Translation of the Schwartz and Lipkus scales

The Japanese version of the Lipkus scale (Lipkus-J), which includes the Schwartz scale (Schwartz-J), was prepared using forward and backward translation [25]. Each translation process was conducted by independent professional translators. Two raters (a bilingual Japanese-English individual and a native English speaker) evaluated the concordance between the back-translated items and the originals. Forward-backward translation was repeated until both raters rated all the back-translated items as semantically concordant with the original. After the translation, some expressions (for example, a lottery prize in dollars) were changed to suit the Japanese context. Finally, the understandability of the resultant wording was checked by students, office workers, and researchers recruited at Jichi Medical University, and Obihiro University of Agriculture & Veterinary Medicine (n = 22), and minor changes in wording were made.

### Other measures

#### Framing bias

A set of questions in which subjects were asked to rate the risk level of a surgical procedure when risk information was presented in two different frames (survival rate, “991 in 1000 people survive this surgery”, and death rate, “9 in 1000 people die from this surgery”), were adopted from the Medical Data Interpretation test [51]. Framing was manipulated within subjects, separating the two differently framed questions with 12 irrelevant ones. A four-point scale was used to rate risk level (1 = not risky, 2 = slightly risky, 3 = risky, 4 = very risky). The framing effect was evaluated by examining the difference between risk rating scores obtained for the two frames.

#### TAIMI measure

As mentioned in the Introduction, TAIMI [22] contains health numeracy items for Japanese adults. To examine how performance on TAIMI relates to that on the numeracy scales used in the current study, TAIMI was included in the survey.

### Survey and participants

An online survey company (Cross Marketing, Tokyo, Japan) was contracted to collect responses (n = 300), and recruitment e-mails were sent to a participant pool maintained by a different online survey company (Research Panel, Tokyo, Japan, n > 1.4 million). Participants voluntarily agreed to complete the online survey. We created 20 blocks of subjects, each defined by gender, age group (20-29, 30-39, 40-49, 50-59, 60-69 years old), and education level (low attainment [high school or lower], high attainment [high school or higher]). The quota was set so that the sample composition roughly matched the Japanese adult population (Additional file 1: Table S1). Participants were recruited until the quota was filled. Students and medical professionals were excluded.

The survey included the Lipkus-J scale (which incorporates the Schwartz-J), TAIMI, measures for framing bias, and some other measures of health and risk attitudes (not reported here). The web page was designed in such a way that respondents could not proceed to the next question without completing the current one, so there were no non-response items in the survey. The survey was conducted in March 2011.

### Statistical analysis

Item responses for Schwartz-J, Lipkus-J, and TAIMI were first dichotomized to be either correct or incorrect, and the percentage of individuals with the correct response was determined for each item. As in original scales, the total score was calculated as the number of correct items for each respondent.

An exploratory factor analysis with binary variables was conducted using Mplus version 6.12 [52]. The method used employs tetrachoric correlation with weighted least squares means and variance adjusted (WLSMV) estimation method. This method accommodates dichotomous observation, and has been indicated to be robust to ceiling effects [53-56].

Numbers of factors of the new scales were determined by parallel analysis. In the analysis, random datasets for the same number of items and participants as actual observation were generated. Eigen values were extracted for each random dataset, and actual observation. Only those factors with eigen values greater than the average eigen values obtained from random datasets were deemed to be meaningful [57]. Subsequently, exploratory factor analysis with number of factors determined by parallel analyses was performed to examine the factor loadings. Criteria for factor loadings were set to be .35 and above [58]. The consistencies of scales were evaluated according to classical test theory, including Cronbach’s alpha [59], item-total correlation, and descriptive statistics of items.

Convergent validity of numeracy scales has been evaluated through correlation with existing measures for health numeracy [8]. We examined correlations between TAIMI scores and Schwartz-J, and Lipkus-J scores. TAIMI has a two-factor structure where three items are especially relevant to numeracy [22]. Therefore, we expected a positive correlation between performance on TAIMI and the scales translated in this study, especially for the numeracy items of TAIMI (TAIMI-num).

Because the assumption of a normal distribution was not satisfied for test statistics, we used non-parametric tests for examining the effects of demographic characteristics on test performance, and of numeracy levels on framing bias. The Wilcoxon signed rank test was used for pair-wise comparison between two conditions. The Mann-Whitney test was used to compare between two groups, and the Kruskal–Wallis test followed by a pair-wise Mann-Whitney test with Bonferroni correction was used to compare between three or more groups. Significance levels were set at p < 0.05. The program, IBM SPSS Statistics 19 was used for most statistical analysis, with M-plus version 6.12 [52] was used for factor analysis.

### Ethical considerations

The institutional ethics committee of the National Food Research Institute granted approval for the study, and permission was also obtained from management section of the Obihiro University of Agriculture & Veterinary Medicine. The methodology used in this study followed the principles of the Helsinki Declaration. Collection of on-line data complied with requirements specified in Japanese Industrial Standards “Personal information protection management systems - Requirements” (JIS Q 15001). Written (electrical) consent was obtained from all the participants.

## Competing interests

The authors declare that they have no competing interests.

## Authors’ contributions

MO designed the study, collected, analyzed, and interpreted the data, and wrote the manuscript. YK designed the statistical analysis procedures, contributed to the study design, supported collection, analysis and interpretation of the data, and helped with the manuscript development. MS and EW contributed to the questionnaire development and data collection. LC contributed to questionnaire development, data analysis, and manuscript development. ID contributed to design the study, collection, analysis, and interpretation of the data, and manuscript development. KK contributed to questionnaire development, data collection and study coordination. All authors have read and approved the final manuscript.

## Pre-publication history

The pre-publication history for this paper can be accessed here:

http://www.biomedcentral.com/1472-6947/12/104/prepub

## Supplementary Material

Additional file 1 **Table S1. **Education levels of the Japanese population. Education histories for different generations and genders. Percentage of people with a high school education or lower in the Japanese adult population for each category is shown based on the latest national survey (Ministry of Internal Affairs and Communications, as of October 1, 2007). Sampling quotas in the current study were allocated according to this proportion. Click here for file

Additional file 2 **Table S2. **Household income of the Japanese population. Percentage of people in each household income category in the Japanese adult population (Population column) and in the current sample (Sample column). Data is based on the latest national survey (Ministry of Internal Affairs and Communications, as of October 1, 2007). Click here for file

Additional file 3 **Table S3. **Numeracy scores for each demographic sub-group. Mean ± standard deviation is shown for each sub-group. Scores between subgroups were compared using non-parametric methods, but means are presented because median scores did not show differences between sub-groups. The effect of gender and educational attainment was significant for both scales (Mann–Whitney's test, effect of gender, Schwartz-J, Z=2.6, p<0.01; Lipkus-J^9^, Z=2.6, p<0.01; effect of education, Schwartz-J, Z=2.0, p<0.05; Lipkus-J^9^, Z=2.3, p<0.05). The effect of age was significant only for Schwartz-J, where post hoc analysis revealed that the 40-49 year old group performed significantly better than the 60-69 year old group (Mann-Whitney's test, Z=2.9, p<0.05, Bonferroni corrected). Click here for file
