# Supplementary material for: Health numeracy in Japan: measures of basic numeracy account for framing bias in a highly numerate population
Source: BMC Med Inform Decis Mak. 2012 Sep 11;12:104. doi: 10.1186/1472-6947-12-104 (PMC3511058; doi:10.1186/1472-6947-12-104)
Supplement: Additional file 1 — Table S1. Education levels of the Japanese population. Education histories for different generations and genders. Percentage of people with a high school education or lower in the Japanese adult population for each category is shown based on the latest national survey (Ministry of Internal Affairs and Communications, as of October 1, 2007). Sampling quotas in the current study were allocated according to this proportion. [file 1472-6947-12-104-S1.doc]

Supplementary Table 1 Education levels of the Japanese population

|  |  |  |  |  |  |  |
| --- | --- | --- | --- | --- | --- | --- |
|  | Age group | | | | |  |
|  | 20-29 | 30-39 | 40-49 | 50-59 | 60-69 | all |
| Female | 37 | 42 | 50 | 66 | 82 | 55 |
| Male | 46 | 46 | 49 | 61 | 74 | 55 |

Education histories for different generations and genders. Percentage of people with a high school education or lower in the Japanese adult population for each category is shown based on the latest national survey (Ministry of Internal Affairs and Communications, as of October 1, 2007). Sampling quotas in the current study were allocated according to this proportion.
