# Supplementary material for: Health numeracy in Japan: measures of basic numeracy account for framing bias in a highly numerate population
Source: BMC Med Inform Decis Mak. 2012 Sep 11;12:104. doi: 10.1186/1472-6947-12-104 (PMC3511058; doi:10.1186/1472-6947-12-104)
Supplement: Additional file 2 — Table S2. Household income of the Japanese population. Percentage of people in each household income category in the Japanese adult population (Population column) and in the current sample (Sample column). Data is based on the latest national survey (Ministry of Internal Affairs and Communications, as of October 1, 2007). [file 1472-6947-12-104-S2.doc]

Supplementary Table 2 Household income of the Japanese population

|  |  | Population |  | Sample |
| --- | --- | --- | --- | --- |
| Household income | < 3 | 23.1 |  | 19.7 |
|  | 3 ≤ 5 | 22.8 |  | 21.3 |
|  | 5 ≤ 8 | 25.9 |  | 32.0 |
|  | ≥ 8 | 28.3 |  | 18.3 |

Percentage of people in each household income category in the Japanese adult population (Population column) and in the current sample (Sample column). Data is based on the latest national survey (Ministry of Internal Affairs and Communications, as of October 1, 2007).
