# Supplementary material for: Health numeracy in Japan: measures of basic numeracy account for framing bias in a highly numerate population
Source: BMC Med Inform Decis Mak. 2012 Sep 11;12:104. doi: 10.1186/1472-6947-12-104 (PMC3511058; doi:10.1186/1472-6947-12-104)
Supplement: Additional file 3 — Table S3. Numeracy scores for each demographic sub-group. Mean ± standard deviation is shown for each sub-group. Scores between subgroups were compared using non-parametric methods, but means are presented because median scores did not show differences between sub-groups. The effect of gender and educational attainment was significant for both scales (Mann–Whitney's test, effect of gender, Schwartz-J, Z=2.6, p<0.01; Lipkus-J9, Z=2.6, p<0.01; effect of education, Schwartz-J, Z=2.0, p<0.05; Lipkus-J9, Z=2.3, p<0.05). The effect of age was significant only for Schwartz-J, where post hoc analysis revealed that the 40-49 year old group performed significantly better than the 60-69 year old group (Mann-Whitney's test, Z=2.9, p<0.05, Bonferroni corrected). [file 1472-6947-12-104-S3.doc]

Supplementary Table 3 Numeracy scores for each demographic sub-group

|  |  | Schwartz-J | Lipkus-J^9^ |
| --- | --- | --- | --- |
| Gender |  |  |  |
|  | Male | 2.5±0.8 | 8.2±1.3 |
|  | Female | 2.3±0.9 | 7.7±1.8 |
| Education |  |  |  |
|  | Low | 2.3±0.9 | 7.8±1.7 |
|  | High | 2.5±0.7 | 8.2±1.3 |
| Age |  |  |  |
|  | 20-29 | 2.4±0.9 | 8.1±1.5 |
|  | 30-39 | 2.4±0.8 | 8.0±1.5 |
|  | 40-49 | 2.6±0.8 | 8.3±1.4 |
|  | 50-59 | 2.5±0.8 | 8.0±1.4 |
|  | 60-69 | 2.2±1.0 | 7.6±1.9 |
| Household income　(million yen, %) | | | |
|  | < 3 | 2.3±1.0 | 7.6±2.0 |
|  | 3 ≤ 5 | 2.3±0.8 | 7.9±1.5 |
|  | 3 ≤ 8 | 2.5±0.8 | 8.2±1.2 |
|  | ≥ 8 | 2.5±0.7 | 8.2±1.6 |

Mean ± standard deviation is shown for each sub-group. Scores between subgroups were compared using non-parametric methods, but means are presented because median scores did not show differences between sub-groups. The effect of gender and educational attainment was significant for both scales (Mann–Whitney's test, effect of gender, Schwartz-J, Z=2.6, p<0.01; Lipkus-J^9^, Z=2.6, p<0.01; effect of education, Schwartz-J, Z=2.0, p<0.05; Lipkus-J^9^, Z=2.3, p<0.05). The effect of age was significant only for Schwartz-J, where post hoc analysis revealed that the 40-49 year old group performed significantly better than the 60-69 year old group (Mann–Whitney's test, Z=2.9, p<0.05, Bonferroni corrected).
